# Engineering the porosity and acidity of H-Beta zeolite by dealumination for the production of 2-ethylanthraquinone *via* 2-(4′-ethylbenzoyl)benzoic acid dehydration†

**DOI:** 10.1039/c7ra13576a

**Published:** 2018-03-08

**Authors:** J. X. Liu, N. He, C. Y. Liu, G. R. Wang, Q. Xin, H. C. Guo

**Affiliations:** State Key Laboratory of Fine Chemicals, School of Chemical Engineering, Dalian University of Technology Dalian 116024 China hongchenguo@dlut.edu.cn +86-411-84986120; State Key Laboratory for Catalysis, Dalian Institute of Chemical Physics, Chinese Academy of Sciences Dalian 116023 Liaoning China

## Abstract

Environmentally-friendly zeolites have been used commercially to replace concentrated sulfuric acid and oleum in the alkylation reactions and dehydration of alcohols. However, moderate activity, associated with access and diffusion limitations, low intramolecular dehydration selectivity, associated with unsatisfactory acidity, and unknown reusability have hampered their industrial implementation in the dehydration of bulky 2-(4′-ethylbenzoyl)benzoic acid (E-BBA) to 2-ethylanthraquinone (2-EAQ). Herein, we have discovered that after being treated with mild HNO_3_, nano-sized H-Beta zeolite showed outstanding catalytic activity, selectivity and reusability, compared with a commercial oleum catalyst. A number of techniques, such as XRD, XPS, XRF, ^29^Si MAS NMR, ^27^Al MQ MAS NMR, FTIR, NH_3_-TPD, argon physisorption and HR-TEM, have been employed to decouple the interdependence between acidity, porosity and catalytic performance. It was found that mild HNO_3_ treatment could clean out the extra-framework aluminium deposits and selectively extract the aluminium species on the outer surface of Beta zeolites, which strengthened the acidity of the Brønsted acid sites (Si(OH)Al) inside the H-Beta micropores, thus increasing the possibility of intramolecular dehydration of E-BBA. Moreover, this mild HNO_3_ treatment also dredged the network of intercrystalline mesopores, alleviating the diffusion constraints. Therefore, through the dual adjustment of acidity and porosity, dealuminated H-Beta zeolite has a promising future in the green synthesis of 2-EAQ.

## Abstract 2

Mild HNO_3_ treated nano-sized H-Beta zeolite showed outstanding catalytic activity, selectivity and reusability, compared with a commercial oleum catalyst.

## Graphical abstract

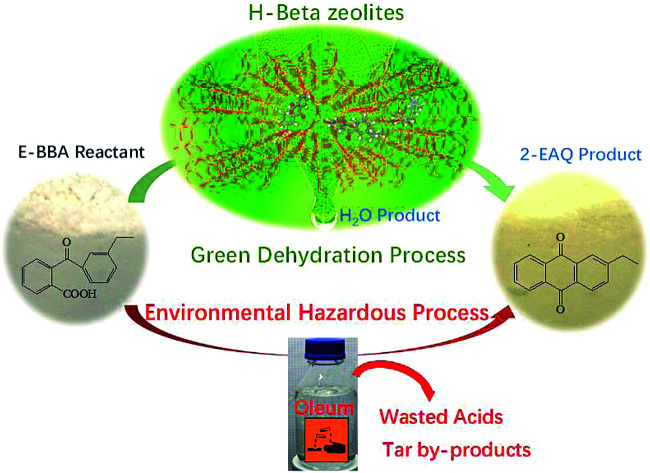

## Introduction

With the issue of environmental protection becoming more and more critical, some pollution technologies in the chemical industries urgently need to be improved or replaced. With 2-ethylanthraquinone (2-EAQ), an important raw material, 90% of it is used in the synthesis of hydrogen peroxide (H_2_O_2_),^1,2^ and the other 10% is used for the synthesis of dye intermediates and photosensitizers.^3^ Hydrogen peroxide (H_2_O_2_) is a highly efficient and green oxidant because it has the highest content of active oxygen (47.1% w/w) and only H_2_O as the by-product. In recent years, commercial H_2_O_2_ has been widely applied in the epoxidation of propylene to produce propylene oxide and the oxidation of cyclohexanone amine to produce cyclohexanone oxime. Nowadays, H_2_O_2_ is manufactured almost exclusively through the anthraquinone process using 2-EAQ for hydrogenation.^3^ The demand for 2-EAQ worldwide in 2016 was more than 20 000 tons and is expected to increase at a rate of 8%.

Three industrialized synthetic routes for producing 2-EAQ have been developed: the phthalic anhydride (PHA) method, the anthracene oxidation method, and the naphthoquinone method.^2^ From the viewpoint of the low production cost, the abundance of the raw materials, and the facile operation process, the PHA method is widely used in industry. The commercially synthetic process includes two steps; in the first step, AlCl_3_ is used as the catalyst for the acylation of ethylbenzene with PHA to produce 2-(4′-ethylbenzoyl)benzoic acid (E-BBA); in the second step, concentrated sulfuric acid or oleum is used as the catalyst for the dehydration of E-BBA to 2-EAQ.^4–7^

Generally, each ton of 2-EAQ product would consume 6.5 tons of oleum, which could result in more than 16 tons of waste acid (about 40 wt% concentration). The waste acid is very difficult to use again and is extremely difficult to be disposed of because it also contains a lot of organic compounds. In addition to such environmental problems, the oleum catalysed E-BBA dehydration also suffers from the formation of a large amount of sticky tar by-products due to heavily intermolecular oligomerization. Consequently the selectivity of 2-EAQ is only 80%. Therefore, under the dual pressure of a fast-growing demand for 2-EAQ and the need for environmental protection, nowadays 2-EAQ producers eagerly desire new environmentally benign and more selective E-BBA dehydration catalysts to replace the concentrated sulfuric acid and oleum catalysts.

Zeolites are well-known solid acid catalysts, which have been used in many industrial processes. Zeolites have many merits such as being highly eco-friendly and having high thermal stability, adjustable acidity and porosity and, more importantly, unique shape-selectivity. Zeolites have already been used to replace liquid acid catalysts such as AlCl_3_·HCl, HF, concentrated sulfuric acid and oleum in ethylbenzene synthesis, cumene synthesis, alkylation, and alcohol dehydration.^8,9^ H-Beta zeolite catalysed E-BBA dehydration has been reported by Xu *et al.*^10^ The study showed that HNO_3_ treatment (concentration: 0.5–5 M, 80 °C, time: 2 h twice, liquid/solid ratio: 10) could enhance E-BBA conversion and the 2-EAQ selectivity of the H-Beta zeolite catalyst. However, little is known about the catalytic chemistry from the limited publications.^11^

In recent years, we have carried out systematic studies on H-Beta zeolite catalysed E-BBA dehydration. In this study, a nano-sized H-Beta zeolite and three kinds of HNO_3_ leached nano-sized H-Beta zeolite were used as catalysts for the dehydration of E-BBA to 2-EAQ. A number of techniques, such as XRD, XPS, XRF, ^29^Si MAS NMR, ^27^Al MQ MAS NMR, FTIR, NH_3_-TPD, argon physisorption and HR-TEM, have been employed to illuminate the relationship between the catalytic properties and the performance. Moreover, density functional theory (DFT) studies were also performed to gain insight into the advantages of H-Beta zeolite. It was found that the mild HNO_3_ leaching treatment is helpful for enhancing both E-BBA conversion and 2-EAQ selectivity. At full conversion of E-BBA, the selectivity of 2-EAQ over mildly treated nano-sized H-Beta was 89%, which is higher than that of a commercial oleum catalyst (80%). Moreover, the zeolite catalyst could be recycled after regeneration at least seven times without a notable degradation in catalytic performance.

## Experimental

### Materials

Nano-sized NH_4_-Beta zeolites (20–50 nm, Fig. S1†) with a Si/Al ratio of 12 were supplied by Dalian Ligong Qiwangda Chemical Technology (DQ-TECH). H-Beta zeolite was obtained from the calcination of NH_4_-Beta zeolite under a dry air flow of up to 540 °C for 6 hours.

Dilute nitric acid leaching was carried out at 85 °C under stirring the zeolite with 0.3 M, 0.6 M and 2.0 M concentrations of HNO_3_. The acid solution to zeolite ratio was 30 mL g^−1^ and the leaching time was 5 h. After leaching, the zeolitic powders were recovered by filtration and washed repeatedly until the pH value was equal to that of deionized water. Then, the filter cakes were dried at 110 °C for 24 h and calcined at 540 °C for 3 h. The calcined samples were labeled as H-Beta(0.3), H-Beta(0.6), and H-Beta(2.0), corresponding to 0.3 M, 0.6 M, and 2.0 M HNO_3_ solutions, respectively.

### Characterization

HR-TEM images of the nano-sized H-Beta zeolites were recorded on a JEOL JEM-2100 (200 kV) microscope.

X-ray diffraction (XRD) patterns were obtained with a Rigaku D/max-2004 diffractometer with Cu Kα radiation (40 kV, 100 mA) and a 0.02° min^−1^ (2*θ*) scanning speed.

X-ray fluorescence (XRF) measurements were performed with a Bruker SRS3400 spectrometer to determine the bulk silicon to aluminum ratio.

X-ray photoelectron spectroscopy (XPS) analyses were conducted with a VG ESCALAB MK2 instrument using Al Kα radiation (1486.6 eV) in order to estimate the surface silicon to aluminum ratio. The voltage and power used for the measurements were 12.5 kV and 250 W, respectively. The vacuum in the test chamber during spectrum collection was maintained at 2 × 10^−10^ mbar. The binding energies were calibrated for the surface charge by referencing to the C_1_S peak of the contaminant carbon at 284.6 eV.

Solid-state MAS NMR measurements were performed with an Agilent DD2-500 MHz spectrometer. ^27^Al MAS NMR spectra were acquired at 130.2 MHz using a 4 mm MAS NMR probe with 14 kHz spinning speed. The chemical shifts of aluminum were referenced to (NH_4_)Al(SO_4_)_2_·12H_2_O at ∼0.4 ppm as a secondary reference. The spectra were accumulated for 200 scans with a π/12 flip angle and 2 s pulse delay. ^27^Al MQ MAS NMR spectra were collected using a three-pulse sequence incorporating a z-filter. A two-dimensional Fourier transformation followed by a shearing transformation gave a pure absorption mode 2D contour plot. The second-order quadrupolar effect (PQ) and isotropic chemical shift (*δ*_iso_) values were calculated according to the procedure introduced in the reference.^15^^29^Si MAS NMR spectra were collected at 99.3 MHz using a 6 mm MAS probe with 4 kHz spinning speed, for 400 scans and with a 4 s pulse delay. 4,4-Dimethyl-4-silapentane sulfonate sodium (DSS) was used as the chemical shift reference for the ^29^Si MAS NMR spectroscopy.

NH_3_-TPD measurements were employed to investigate the overall acidity of the catalysts. Profiles were obtained with a Quantachrome ChemBet 3000 chemisorb instrument. Samples (150 mg, 380–830 μm sieve fraction) were pretreated in He at 600 °C for 1 h and then cooled down to 100 °C for ammonia adsorption. The ammonia adsorption was carried out at 100 °C for 30 minutes with a mixture of 5% NH_3_ in He. After the adsorption the cell was purged in 50 mL min^−1^ He flow for 30 min to remove all non-chemically adsorbed NH_3_. Then the NH_3_-TPD profiles were recorded in a 50 mL min^−1^ He flow by ramping the temperature from 100 to 600 °C at a rate of about 16 °C min^−1^.

Fourier transform infrared spectroscopy (FTIR) was also used to characterize the acidity of the H-Beta zeolite samples. The spectra for surface hydroxyl (OH) vibrations and pyridine adsorption were obtained with a Nicolet is10 FT-IR spectrometer. The zeolitic samples were pressed into a self-supporting thin wafer (approximately 15 mg) and decontaminated at 400 °C under vacuum (10^−3^ Pa) for 4 h in a quartz IR cell equipped with CaF_2_ windows. After the pretreatment, the cell was cooled down to RT for sample measurement. Spectra were recorded from 4000 to 400 cm^−1^ with an optical resolution of 4 cm^−1^. The hydroxyl vibration spectra were obtained by subtracting the background spectrum (recorded with an empty IR cell in the absence of sample) from the measured sample spectra. The spectra for pyridine were obtained as follows: first, pyridine adsorption was carried out at 150 °C for 10 minutes in pyridine vapor (approximately 2.2 mbar). Then, evacuation treatment (10^−3^ Pa) was conducted for 30 minutes at 150 °C. The spectra were obtained by subtracting the background spectrum (obtained with decontaminated wafers before pyridine adsorption) from the measured sample spectra.

Argon physisorption was conducted on a Micromeritics ASAP 2020 instrument at 87 K to obtain textural information. Prior to measurement, the samples (380–830 μm sieve fraction) were degassed at 623 K for 6 h. The surface area was calculated using the Brunauer–Emmett–Teller (BET) method using the adsorption branch in the *p*/*p*_0_ range from 0.10 to 0.15: the pore volumes were estimated at *p*/*p*_0_ of 0.99, while micro- and mesoporosity was discriminated using the *t*-plot method.

### Catalytic tests

Dehydration of 2-(4′-ethylbenzoyl)-benzoic acid (E-BBA) was performed under atmospheric pressure in a three-necked flask. The temperature of the reaction was maintained using an electric heater. In a typical experiment, 2.0 g E-BBA was loaded into the flask and heated to melt with the solvent, to which 2.0 g H-Beta zeolite powder catalyst was added whilst stirring, then the mixture was heated to reaction temperature and the reaction carried out at a given temperature for 3.5 h with vigorous stirring. After the reaction, the mixture in the flask was cooled down to room temperature and dissolved in 1,4-dioxane. The zeolite catalyst was removed by filtration, washed with 1,4-dioxane, dried in an oven at 90 °C, and further regenerated by calcining it in air at 500 °C for 3 h if necessary. The filtrate was analyzed using a liquid-chromatograph (Waters 1525) equipped with a SunFire C18 column with a particle size of 5 μm and dimensions 150 × 4.6 mm. The mobile phase was a mixture of H_2_O, CH_3_OH and tetrahydrofuran (THF), flowing at a rate of 0.5 mL min^−1^, with HPLC spectra collected at a UV wavelength of 274 nm. The calibration curves were linear for E-BBA (*r* = 0.9995) and 2-EAQ (*r* = 0.9997). The weights of E-BBA and 2-EAQ were calculated based on their peak areas and the correction factors. The liquid was analyzed by atmospheric pressure ionization electrospray mass spectrometry (API-ES-MS) with a HP1100LC/MSD mass spectrometer.

### DFT calculations

**Fig. 1:**
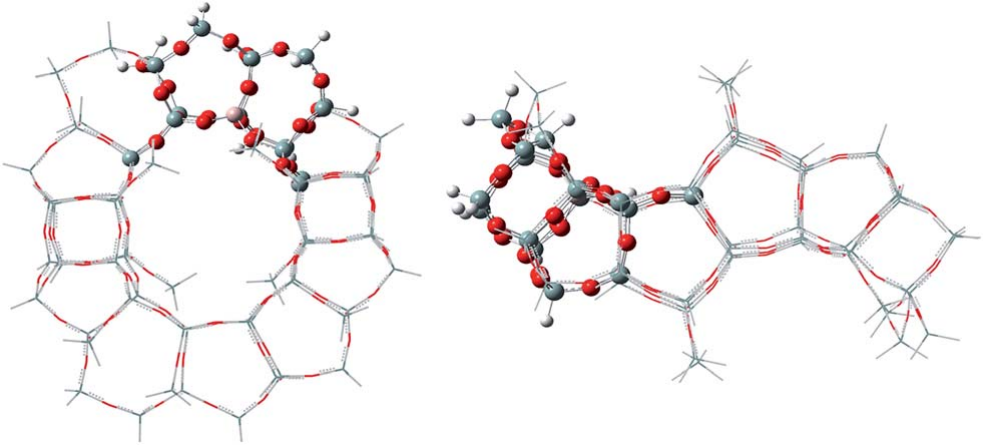
The 72T ONIOM cluster (the atoms treated with ωB97XD and AM1 are shown in ball and stick modelling and the wireframe, respectively).

A 72T cluster model with 247 atoms and the Al atoms placed in the T6 lattice positions was obtained by cutting the periodic structure, as shown in Fig. 1. The dangling silicon atoms are terminated by substituting the lattice O atoms with H atoms, giving Si–H bond lengths of 1.46 Å. To improve the energetic properties and take into account the effect of the entire zeolite framework on the reaction mechanism, a two-layer scheme ONIOM was employed. Therefore, the regions related to the catalytic reactions were treated at high-level with the functional ωB97XD along with the 6-31+G(d,p) basis set for accuracy, which is a long-range corrected functional.^12^ The regions away from the active center were treated at lower-level with the semi-empirical calculation method (AM1) for efficiency. During all calculations, the positions of terminal Si–H groups in the cluster model were held fixed in their crystallographic positions to retain the zeolite structure, whereas the positions of the remaining atoms and reactant molecule were optimized. The transition state structures were characterized by means of frequency calculations with only one imaginary frequency. The intrinsic reaction coordinate (IRC) method was used when necessary to identify the two minima connected by a transition state. In addition, the acidity of the E-BBA molecule and H-Beta zeolites was calculated using NH_3_ as the probe molecule.

## Results and discussion

### Chemistry of E-BBA dehydration to 2-EAQ

**Scheme 1:**
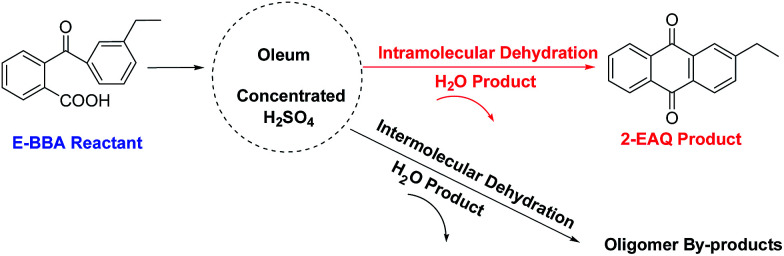
Reaction paths of E-BBA dehydration over oleum and concentrated H_2_SO_4_ catalysts.

As shown in Scheme 1, the dehydration of E-BBA over concentrated sulphuric acid and oleum as catalysts has two possible reaction paths: (1) intramolecular dehydration to 2-EAQ; (2) intermolecular dehydration to oligomer by-products. Owing to the lack of shape-selectivity of oleum as a catalyst, the selectivity of 2-EAQ is not ideal, because of the large amount of sticky tar by-products produced through the heavily intermolecular oligomerization.

**Fig. 2:**
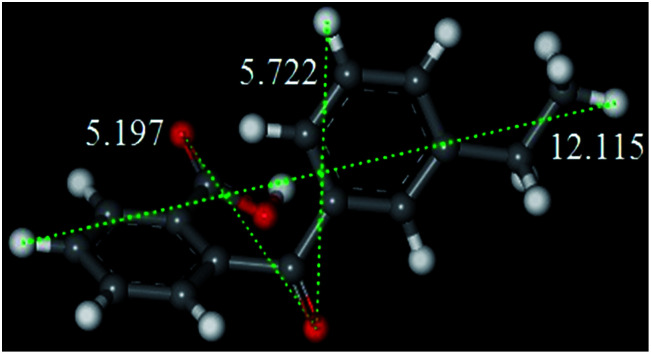
Molecular length of the E-BBA molecule measured from different directions (Å) (the white balls are H atoms, the red balls are O atoms and the grey balls are C atoms).

Beta zeolite possesses a three-dimensional 12-ring pore structure constituted of perpendicular straight channels (6.6 × 6.7 Å) and a sinusoidal channel (5.6 × 5.6 Å).^9^ Compared with the molecular size of E-BBA (shown in Fig. 2), these dimensions mean that the E-BBA molecule can only enter the channel of Beta zeolite in a parallel position (0.58 nm), which is favourable for intramolecular dehydration. However, owing to spatial confinement, intermolecular dehydration between two E-BBA molecules does not happen easily inside the zeolite channel. For H-Beta zeolite, most of the active sites are located inside the microporous channels of the zeolite. Therefore, the selectivity of E-BBA intramolecular dehydration over H-Beta zeolite after precise modification is expected to be higher than that over oleum and concentrated H_2_SO_4_, because of the shape-selective catalysis.

**Scheme 2:**
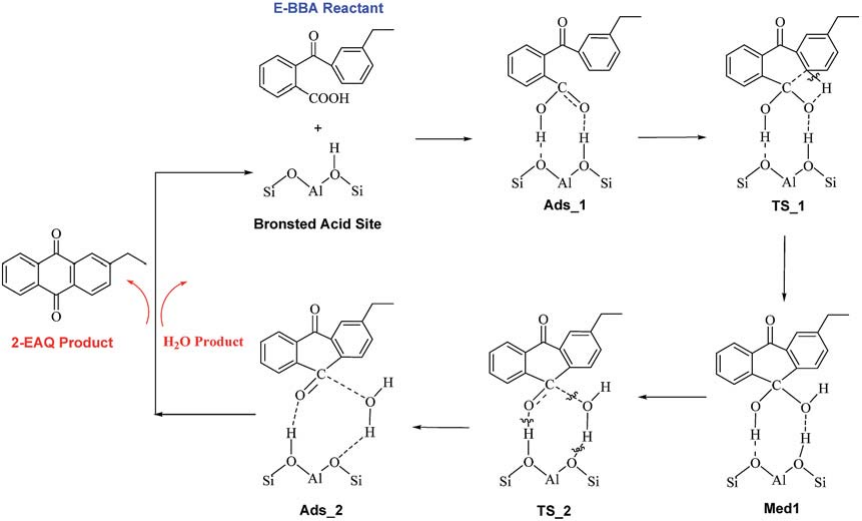
The mechanism of intramolecular dehydration of E-BBA to 2-EAQ over the Brønsted acid sites of H-Beta zeolites.

**Fig. 3:**
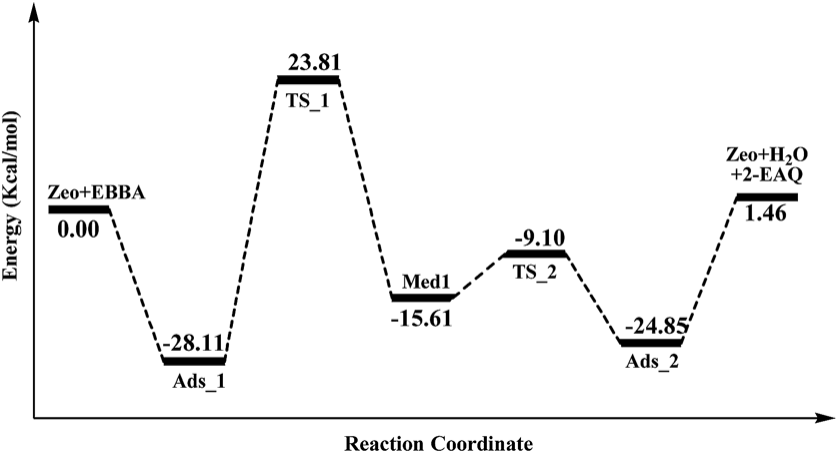
The potential energy surface of the active site (Brønsted acid site) of H-Beta zeolites. The detailed structures of the zeolite and molecules are shown in Fig. S2.†

In order to gain insight into the active site of H-Beta zeolite for E-BBA intramolecular dehydration to 2-EAQ, DFT calculations were performed to obtain the potential energy surface for E-BBA intramolecular dehydration over the Si(OH)Al acid sites. The reaction path is shown in Scheme 2; this path started with physical adsorption, underwent a C–H bond dissociation step, and ended with the desorption of 2-EAQ and H_2_O as products. As seen from Fig. 3, the formation of the O–H bond takes place between the C=O group of the E-BBA molecule and H^+^ on H-Beta zeolite. Then the adsorbed molecule has to go through a transition state (TS_1) by means of H transfer from the C–H bond of ethylbenzene to the O atom of C=O, and at the same time there is formation of a C–C bond. Obviously, the dissociation of a C–H bond and formation of a C–C bond in molecular E-BBA is the rate determining step of the whole reaction due to the high intrinsic activation energy (Δ*E* = 23.81 − (−28.11) = 51.92 kcal mol^−1^). This indicates that the acidity of the catalyst is critical for this reaction. Therefore, the acid strength of the E-BBA molecule and the different hydroxyl groups over H-Beta zeolite were determined by calculating the adsorption energies of NH_3_ as a probe molecule on different hydroxyl groups. As listed in Table 1, the framework aluminium species at different T-positions of H-Beta zeolite have similar acid strengths. The acid strength of the E-BBA molecule and different hydroxyl groups follows the sequence Si(OH)Al > E-BBA > SiOH. This indicates that the framework aluminium species with strong Brønsted acidity could be the effective active sites in E-BBA intramolecular dehydration.

**Table 1:** Adsorption energies of NH_3_ as a probe molecule on different hydroxyl groups of H-Beta zeolites and the E-BBA molecule

	Si(OH)Al (Al at T1–T2)	Si(OH)Al (Al at T3–T9)	SiOH	E-BBA
Δ*E* (kcal mol^−1^)	−30.59	−32.71	−3.47	−15.21

1 MOH + NH_3_ → MOH⋯NH_3_

2 Δ*E*_ads_ = *E*_(MOH⋯NH_3_)_ − (*E*_MOH_ + *E*_NH_3__)

### The effects of dealumination on the composition of H-Beta zeolites

To the best of our knowledge, two major challenges existed for the large, organic molecule E-BBA’s dehydration to 2-EAQ over H-Beta zeolites: (1) strong transportation limitations and (2) low intramolecular dehydration selectivity. In order to overcome these challenges, commercial nano-sized H-Beta zeolite was chosen as the parent catalyst and HNO_3_ leaching treatment was applied to adjust the distance between two neighboring Brønsted acid sites (Si(OH)Al) to suppress undesired intermolecular oligomerization.

As shown in Fig. S3,† the XRD patterns of all samples were obtained. Compared with those of the parent H-Beta zeolite, the XRD patterns of all dealuminated H-Beta zeolites have no obvious change, indicating that there was no significant structure damage after acid leaching. The effects of HNO_3_ treatment on the composition of nano-sized H-Beta zeolite were investigated through XRF and ^29^Si MAS NMR spectroscopy (Fig. S4†) to obtain the total and framework Si/Al ratios, respectively. And XPS analysis was applied to determine the Si/Al ratio on the outer surface of the prepared samples.

As shown in Table 2, an obvious increase in Si/Al ratios can be observed after HNO_3_ treatment. The Si/Al ratios of dealuminated H-Beta zeolites increased with increasing HNO_3_ concentration. For the parent H-Beta zeolite, the total and framework Si/Al ratios were 12 and 22, respectively, indicating the existence of extra-framework aluminum (EFAL) species. After 0.3 M HNO_3_ treatment, the total and framework Si/Al ratios were 30 and 32, respectively, indicating that most of the aluminium species located in the framework of the zeolite and most of the EFAL species had been cleaned out by this mild acid leaching. At the same time, the surface Si/Al ratio obviously increased to 112, suggesting that the aluminium species in the HB0.3 catalyst are framework aluminium, located inside the zeolite channel. However, during the acid treatment with higher concentration, severe extraction of framework aluminium species occurred as the framework Si/Al ratio increased faster than the total Si/Al ratio.

**Table 2:** Textural properties of H-Beta and dealuminated H-Beta zeolite

Cat	Si/Al^a^	Si/Al^b^	Si/Al^c^
HB	12	22	59
HB0.3	30	32	112
HB0.6	63	83	136
HB2.0	163	185	242

a Molar Si/Al ratio determined by XRF.

b Molar Si/Al ratio determined by ^29^Si MAS NMR spectroscopy.^13^

c Molar Si/Al ratio determined by XPS.^14^

**Fig. 4:**
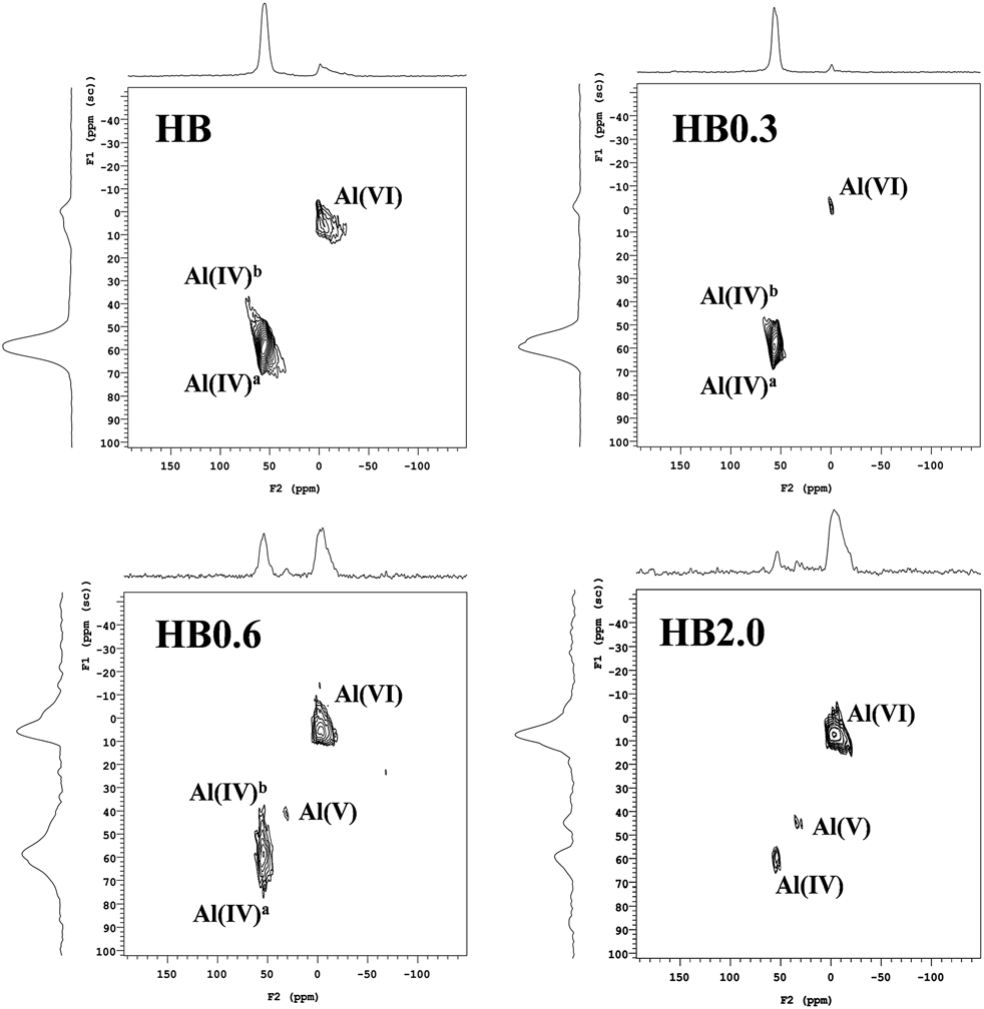
^27^Al MQ MAS NMR spectra of H-Beta and dealuminated H-Beta zeolites. The corresponding ^27^Al MAS NMR spectrum is given on top of the MQ MAS plot. The F1 projection shows a purely isotropic dimension, showing a clear resolution of the resonances corresponding to tetrahedral aluminum at different T-positions.

To obtain deep insight into the effects of acid leaching on Al distribution, ^27^Al MQ MAS NMR spectra of H-Beta and dealuminated H-Beta zeolites were obtained, and the ^27^Al MAS NMR spectra are shown on top of the MQ MAS spectra in Fig. 4. For H-Beta zeolite, two kinds of aluminium species were visible. The resonances corresponding to framework aluminium species in the region 50–60 ppm were assigned to aluminium species at different T-sites in the zeolite called Al(iv)_a_ and Al(iv)_b_. The resonances corresponding to EFAL species were visible in the region −20 to 20 ppm.^15–20^ For HB0.3, the main aluminium species observed were framework aluminium and most of the EFAL species had been removed. The intensity and resonances of Al(iv)_a_ and Al(iv)_b_ were clearly discriminated at 54 and 57 ppm, respectively, suggesting that the acid leaching had removed part of one kind of framework aluminium species so that the two signals become optimal in terms of resolution.^20^ In line with the XRF, ^29^Si MAS NMR and XPS results, it was deduced that the mild HNO_3_ treatment had selectively removed the EFAL species and aluminium species on the external surface of the zeolite.

As the HNO_3_ concentration increased from 0.6 M to 2.0 M, the decrease of the resonance intensity of Al(iv) was accompanied by the resonance intensity of Al(vi) increasing and a new resonance at 30 ppm attributed to penta-coordinated aluminium Al(v) appeared.^19^ As deduced from the XPS, ^29^Si MAS NMR and XRF results, the high HNO_3_ concentration led to the extraction of framework aluminium species to form EFAL species (EFAL). During this process, the penta-coordinated aluminium existed as the transition species for the extraction of framework aluminium.

### The effect of dealumination on the acidic properties of H-Beta zeolites

**Fig. 5:**
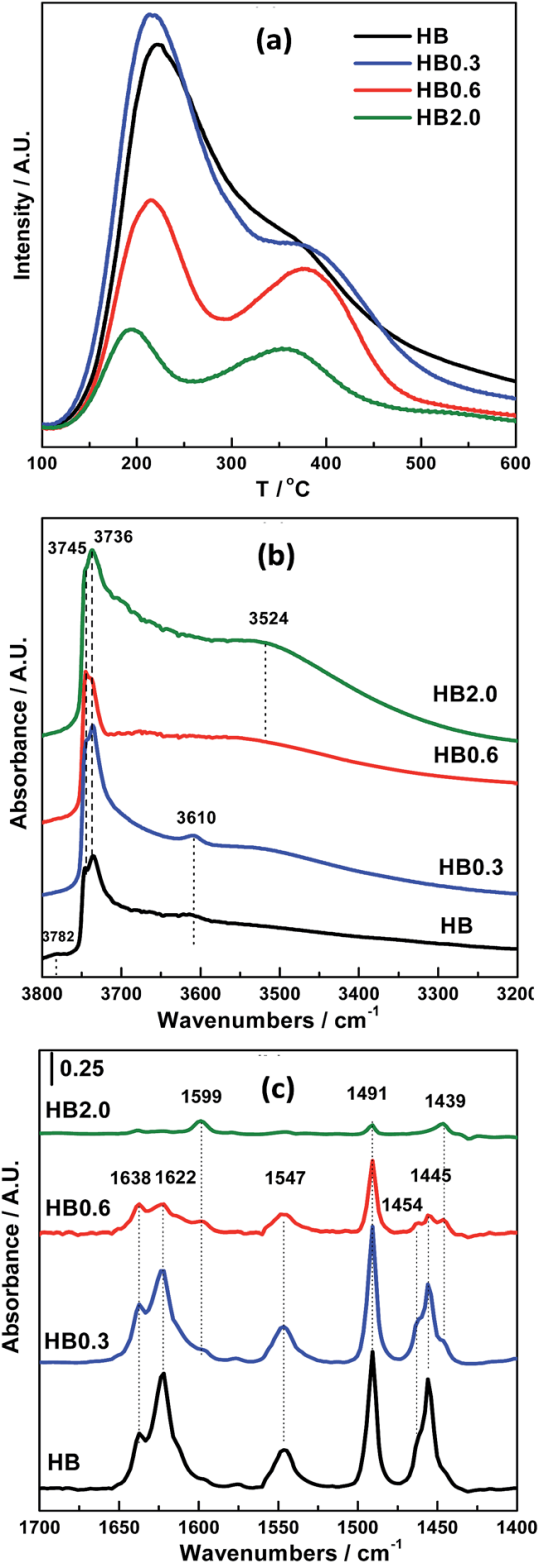
Acidic properties of H-Beta and dealuminated H-Beta zeolites: (a) NH_3_-TPD profiles, (b) OH-IR profiles and (c) Py-IR profiles.

The effect of acid leaching on the acidic properties of H-Beta was studied using NH_3_-TPD and OH-IR and pyridine-IR spectroscopy. Based on the NH_3_-TPD profiles shown in Fig. 5(a), 0.3 M HNO_3_ treatment did not change the total number of acid sites very much, and just shifted the desorption peak at high temperature to a higher temperature. However, for HB0.6 and HB2.0, both the number and strength of the acid sites obviously decreased.

For H-Beta zeolite, four bands can be clearly distinguished in the spectral range of hydroxyl group stretching modes (Fig. 5(b)); the most intense bands at 3736 cm^−1^ and 3745 cm^−1^ are asymmetric and correspond to isolated internal SiO–H groups and isolated external SiO–H groups, respectively.^21–25^ The band at 3610 cm^−1^ is commonly assigned to the bridging hydroxyl groups (Si–(OH)–Al), which cause strong Brønsted acidity. The band at 3782 cm^−1^ is commonly assigned to the AlO–H groups in which Al atoms belong to the extra framework aluminum (EFAL) species. Acid treatment with 0.3 M HNO_3_ resulted in the disappearance of the band at 3782 cm^−1^, indicating that the EFAL species are very sensitive to acid treatment. Furthermore, the obvious increase in the intensity of the bands assigned to bridging hydroxyl groups (3610 cm^−1^) and the silanol bands (3745 cm^−1^ and 3736 cm^−1^), accompanied by the appearance of the band between 3400 and 3600 cm^−1^ (attributed to hydrogen-bonded hydroxyl groups), both indicate the partial extraction of framework aluminium species.^25^ As the HNO_3_ concentration increased to 0.6 and 2.0 M, the intensity of the band between 3400 and 3600 cm^−1^ increased more obviously, at the expense of that at 3610 cm^−1^, suggesting that a substantial amount of framework aluminium species had been extracted.

The same phenomenon was also observed in the pyridine-IR results (Fig. 5(c)). With 0.3 M HNO_3_ treatment of H-Beta zeolite, the number of Lewis acid sites (the bands at 1454 and 1622 cm^−1^), most of which are attributed to EFAL, decreased. In contrast, the number of Brønsted acid sites (the band at 1547 cm^−1^), which are attributed to framework Al, was well preserved. As the concentration of acid increased to 0.6 and 2.0 M, the number of Brønsted and Lewis acid sites simultaneously decreased significantly. In line with the above results, acid treatment using different concentrations obviously has different effects on the acidity of different catalysts. For HB0.3, the acid strength of the Brønsted acid sites (Si(OH)Al) located inside the zeolite channels was strengthened by cleaning out the EFAL species and aluminium species on the external surface of the zeolite. Meanwhile for HB0.6 and HB2.0, the cleanout of the aluminium species was more extensive than this, resulting in a decreased number and strength of the acid sites.

### The effect of dealumination on the porosity of H-Beta zeolites

**Fig. 6:**
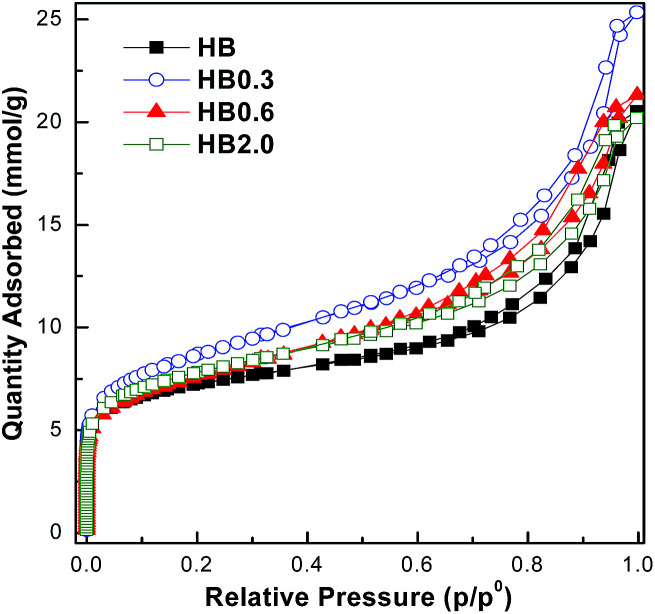
High-resolution Ar adsorption/desorption isotherms at −186 °C.

The theoretically calculated molecular sizes of both intra- and inter-molecular dehydration products of E-BBA (Scheme S1†) were compared with the crystallographic micropore diameters of H-Beta zeolite (Table S1†), and it is easy to see that the shape-selective function of the porous channel of H-Beta zeolite plays a crucial role in crippling the inter-molecular dehydration of E-BBA. The direct comparison of these sizes indicates that the micropores of H-Beta zeolite can exclude most of the formation of inter-molecular dehydration products inside the zeolite channel. Thus, the microporosity of catalysts is also an important factor determining the catalytic performance in this reaction. High-resolution Ar physisorption, an effective method to study the microporosity of zeolites, was employed here. From Fig. 6 it can be seen that all four samples showed a sorption isotherm of type IV with similar hysteresis loops. Compared with that for the parent sample HB, the amount of adsorption increased for all treated samples, but the increase in the degree of adsorption was greater for lower concentrations of acid, *viz.*, HB0.3, HB0.6, and HB2.0, which indicates that mild acid treatment may dredge the channel of zeolites. To get some more details about the pore structure change, the pore size distributions estimated from the adsorption branch of the isotherms using an NLDFT method were investigated in the following.

**Fig. 7:**
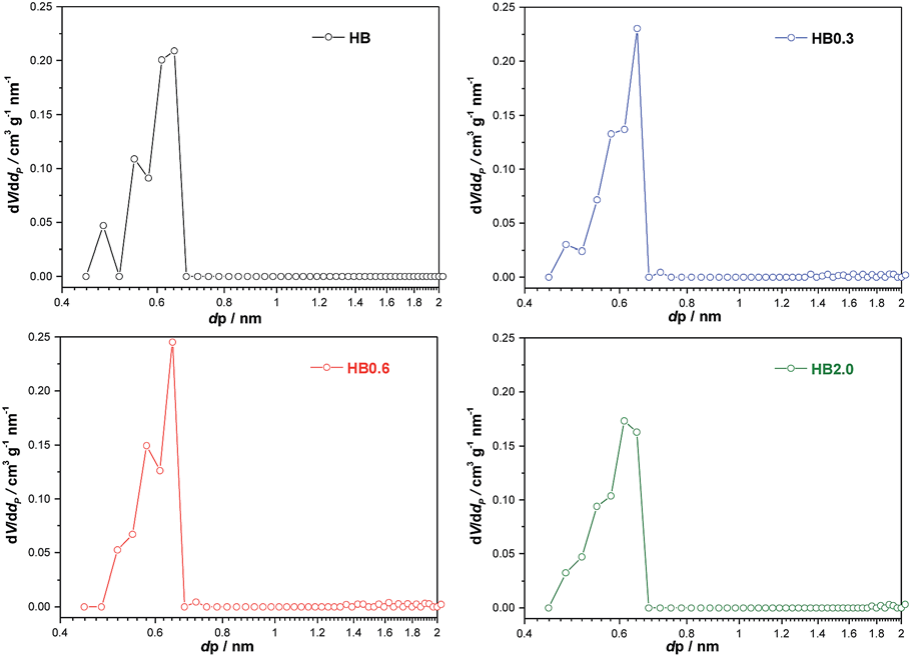
Pore size distributions of H-Beta and dealuminated H-Beta zeolites obtained by application of the NL-DFT method to the Ar adsorption isotherm at −186 °C in the range 0.4–2 nm.

**Fig. 8:**
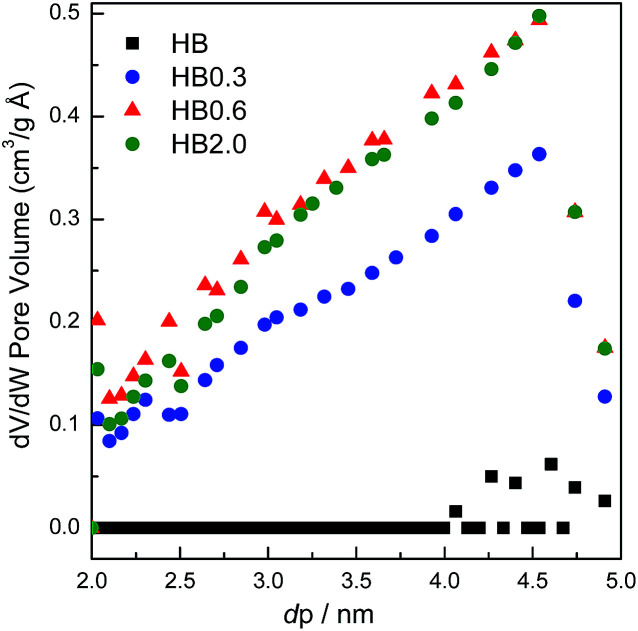
Selected region of the mesopore size distribution of H-Beta and dealuminated H-Beta zeolites.

For the parent sample HB in Fig. 7, there are two main peaks centered at 0.56 nm and 0.65 nm. Also, a small peak centered at 0.48 nm was observed. The existence of micropores with a diameter of 0.48 nm could possibly originate from the blocking of the microporous channels of Beta zeolites by EFAL species. These small micropores disappeared after 0.3 M HNO_3_ treatment. In correlation with XRF and NMR results, the EFAL deposits blocking the micropores were cleared away by this mild HNO_3_ treatment, thus resulting in the increased Ar adsorption amount. On further increasing the HNO_3_ concentration to 0.6 M and 2.0 M, the Ar adsorption volume of the HB0.6 and HB2.0 catalysts began to decrease. According to previous results, the removal of a fraction of framework Al species leads to the formation of EFAL species and the condensation of these species blocked the micropores of zeolite again. Moreover, as shown in Fig. 8, the mesopores, with diameters between 2 and 5 nm, were generated after HNO_3_ leaching. Due to the small single-crystal size of the nano-sized H-Beta parent (20–50 nm), most of these mesopores could possibly be identified as intercrystalline mesopores, which were blocked by EFAL species before. However, the possibility of the existence of intracrystalline mesopores cannot be excluded.

**Fig. 9:**
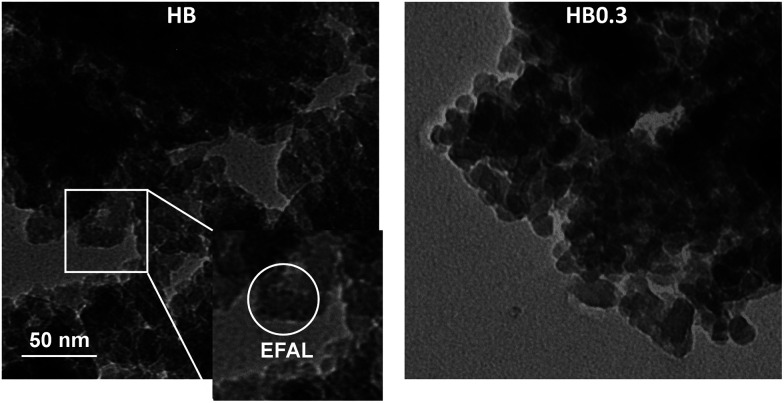
TEM images of H-Beta and dealuminated H-Beta zeolites.

**Fig. 10:**
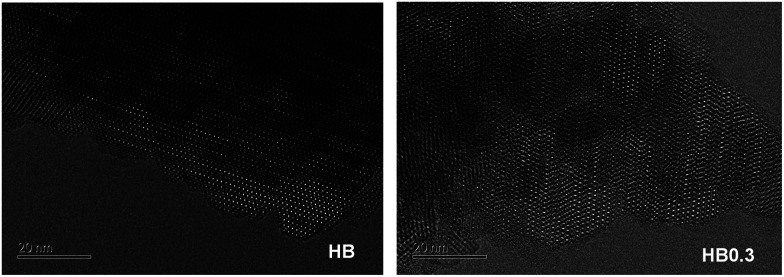
HRTEM images of H-Beta and dealuminated H-Beta zeolites.

High-resolution transmission electron microscopy (TEM) provides insight into the local structure of crystalline phases. Consistent with previous results, TEM spectra gave a direct view (Fig. 9) of 0.3 M HNO_3_ treatment having cleared away the EFAL species. Moreover, the HR-TEM spectra (Fig. 10) have also shown that this mild HNO_3_ treatment did not influence the porous structure of H-Beta zeolite. Therefore, the crucial role of 0.3 M HNO_3_ treatment is that of washing the EFAL deposits and dredging the second network of pores between zeolite nano-crystals, leading to better accessibility of micropores. However, if further increasing the concentration of HNO_3_ to 0.6 and 2.0 M, even though the acid treatment can also clear the channels of the zeolite, some framework Al species were transformed into EFAL deposits, which blocked the microporous channels of the zeolite to some extent, leading to decreased Ar adsorption compared to that of the HB0.3 catalyst.

### The effect of dealumination on nano-sized H-Beta zeolites in the dehydration of E-BBA

**Fig. 11:**
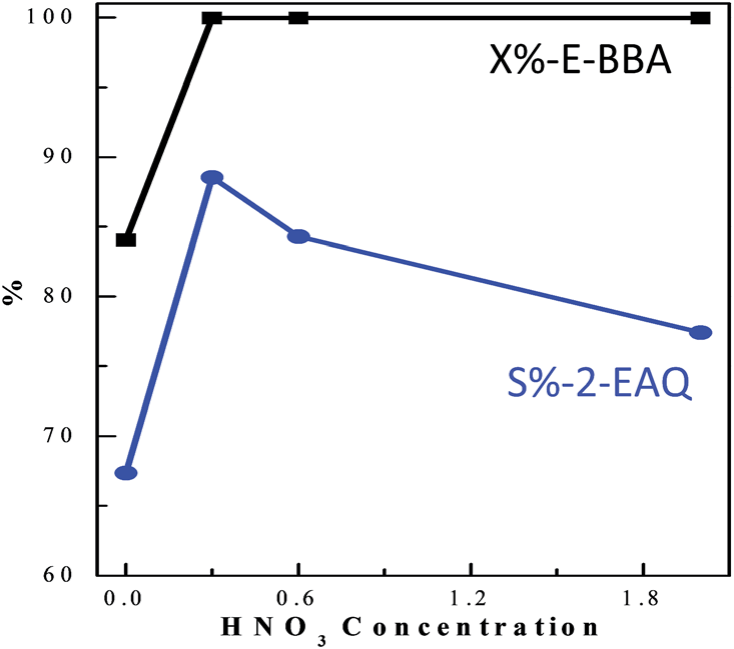
The effect of HNO_3_ treatment on nano-sized H-Beta zeolite in E-BBA dehydration. Reaction conditions: m(E-BBA)/m(Cat) = 1.0, 210 °C, 3.5 h and 0.1 MPa.

**Fig. 12:**
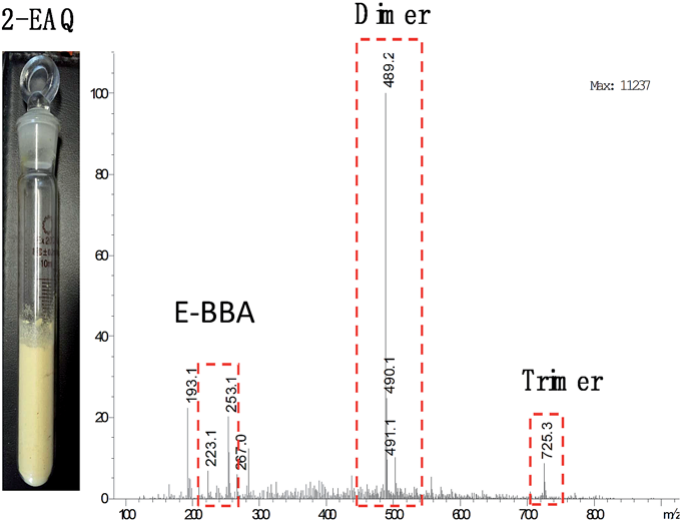
Product analysis for E-BBA dehydration over HB0.3 catalyst. 2-EAQ products obtained by NaOH recrystallization (left) and the API-ES mass spectrum of the washing liquor (right).

**Fig. 13:**
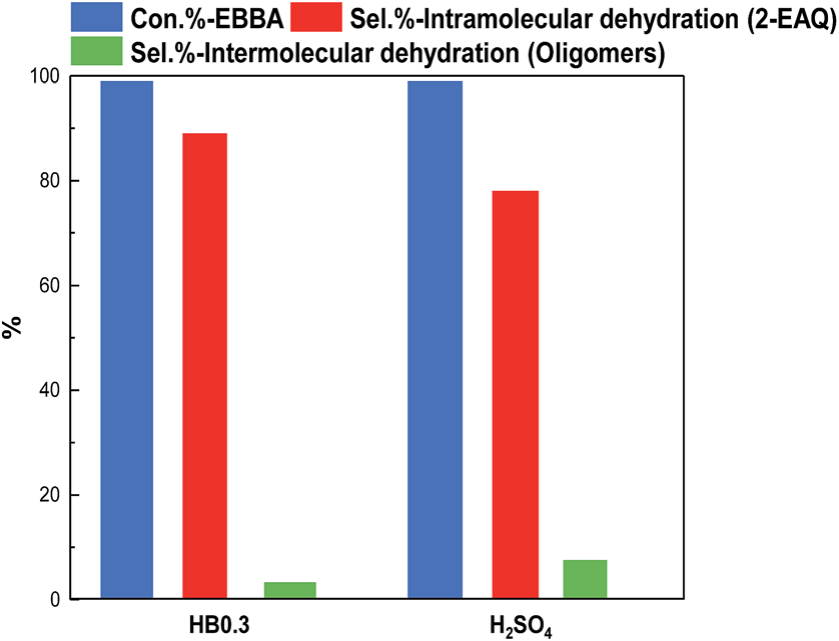
Comparison of HB0.3 with an oleum catalyst for the dehydration of E-BBA. Reaction conditions for HB0.3 catalysed reaction: temperature 210 °C, pressure 0.1 MPa, catalyst loading 2.0 g, E-BBA dosage 2.0 g and reaction time 3.5 h; reaction conditions for oleum catalysed reaction: temperature 98 °C, pressure 0.1 MPa, catalyst loading 9.6 g, E-BBA dosage 2.0 g and reaction time 2 h.

E-BBA dehydration over H-Beta and dealuminated H-Beta zeolites was studied and their performance was compared with that of a commercial oleum catalyst. Nano-sized H-Beta zeolite could catalyze the dehydration of E-BBA but the activity and 2-EAQ selectivity were unsatisfactory (Fig. 11). After 0.3 M HNO_3_ treatment of the H-Beta zeolites, the catalytic performance improved greatly. The E-BBA conversion and 2-EAQ selectivity increased to 99% and 89%, respectively. Further raising the HNO_3_ solution concentration to 0.6 M and 2.0 M had little influence on E-BBA conversion but brought down the 2-EAQ selectivity to 84% and 76%, respectively. In order to obtain insight into this reaction, detailed product analysis was performed. Taking the products over the HB0.3 catalyst as examples, the main product 2-EAQ was first collected through recrystallization and its purity was higher than 98.5%, determined by comparison with a standard reagent. Then the leftover product was further analyzed through API-ES mass spectrometry analysis (shown in Fig. 12). It was found that there were small amounts of unreacted E-BBA and side-products of dimer and trimer produced by the intermolecular dehydration of E-BBA. The selectivity for total oligomers is only around 3% and no formation of larger molecules was found, indicating that the HB0.3 catalyst is more effective in the suppression of heavily intermolecular dehydration (Fig. 13), compared with the high selectivity for sticky tar, 10%, with oleum as catalyst. Thus, the HB0.3 catalyst exhibited better performance than a commercial oleum catalyst in suppressing the formation of large molecular oligomers.

**Fig. 14:**
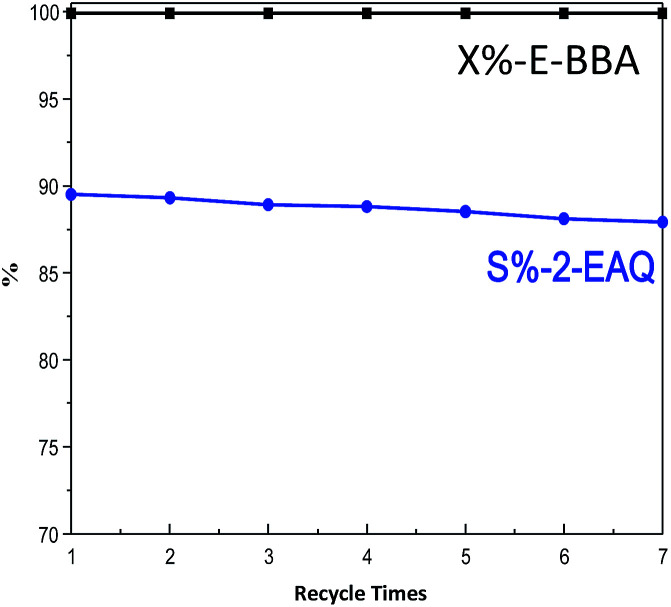
Reusability of the HB0.3 catalyst after regeneration for E-BBA dehydration. Reaction conditions: temperature 210 °C, pressure 0.1 MPa, m(E-BBA)/m(HB0.3 catalyst loading) = 1.0, and reaction time 3.5 h.

Besides the catalytic activity, the reusability of catalysts is also an important issue for industrial application. So, the used HB0.3 catalyst was recovered by facile regeneration in flowing air at 540 °C for 3 hours. The loss of some catalysts is usually inevitable during the regeneration process. Thus, in order to make sure of comparability between the fresh and regenerated catalysts, the mass ratio of E-BBA to HB0.3 catalyst was always maintained at 1 for each test. As seen in Fig. 14, the E-BBA activity and 2-EAQ selectivity remain constant even after seven cycles of regeneration. HB0.3 catalyst showed another significant advantage in the E-BBA dehydration reaction when compared with non-recyclable oleum. The treated Beta zeolites have a promising future in the green synthesis of 2-EAQ *via* E-BBA dehydration.

Therefore, in line with previous characterization results and reaction data, it was found that the low E-BBA conversion over the parent Beta zeolite could be attributed to the blockage of a substantial amount of micropores and intercrystalline mesopores by EFAL species, resulting in difficult accessibility of the E-BBA reactant to Brønsted acid sites located inside zeolite channels. And owing to a lack of space restriction, aluminium species on the outer surface of the parent Beta zeolite are responsible for the intermolecular dehydration of E-BBA to dimer and trimer by-products. Dilute HNO_3_ treatment (0.3 M) could clean out the extra-framework aluminium deposits and selectively extracted the aluminium species on the outer surface of H-Beta zeolite, which not only suppressed the intermolecular dehydration but also strengthened the acidity of the Brønsted acid sites (Si(OH)Al). Moreover, this mild HNO_3_ treatment also dredged the network of intercrystalline mesopores, alleviating the diffusion constraints. Thus, the HB0.3 catalyst exhibited better performance in both E-BBA conversion and 2-EAQ selectivity than the HB catalyst. However, when further increasing the HNO_3_ concentration to 0.6 and 2.0 M, the E-BBA conversion was not influenced but the 2-EAQ selectivity decreased to some extent. As deduced from NMR and Ar physisorption results (Table 2 and Fig. 4 and 8), some of the framework aluminium species were extracted from the zeolite framework lattice generating a large amount of mesopores with diameters between 2 and 5 nm. It is reasonable to deduce that most of these newly formed EFAL species were located in these mesopores, which is different from the location of EFAL species in the parent H-Beta zeolite. The latter is mainly located in micropores and intercrystalline mesopores. The diffusion of E-BBA and 2-EAQ products in the HB0.6 and HB2.0 catalysts is faster than that in H-Beta zeolite. And the accessibility of the acid sites inside the channels of these two catalysts is sufficient to maintain high E-BBA conversion, even though the strength and number of Brønsted acid sites in HB0.6 and HB2.0 apparently decreased (Fig. 5(b) and (c)). These remaining acid sites are sufficient for E-BBA conversion, but the existence of framework vacancies provides a larger space for the intermolecular dehydration of E-BBA, leading to decreased 2-EAQ selectivity.

In summary, the framework aluminium species with strong Brønsted acidity located inside the microporous channels were the main active sites for intramolecular dehydration of E-BBA over H-Beta zeolites to 2-EAQ. Meanwhile the intermolecular dehydration of E-BBA to oligomer by-products is catalysed by the aluminium species on the outer and mesoporous surface of H-Beta zeolites. The diffusion of reactant and product in the zeolite channels is another factor in determining the activity of E-BBA transformation over H-Beta zeolites.

## Conclusions

The catalytic performance of H-Beta and dealuminated H-Beta zeolites in E-BBA dehydration to 2-EAQ has been systematically studied. It was found that after being treated by 0.3 M HNO_3_, nano-sized H-Beta showed outstanding catalytic activity, selectivity and reusability, compared with a commercial oleum catalyst. The mild HNO_3_ treatment could clean out the extra-framework aluminium deposits and selectively extract the aluminium species on the outer surface of H-Beta zeolite, which not only inhibited intermolecular dehydration but also strengthened the acidity of the Brønsted acid sites (Si(OH)Al), thus increasing the possibility of intramolecular dehydration of E-BBA. Moreover, this mild HNO_3_ treatment also dredged the network of intercrystalline mesopores, alleviating the diffusion constraints. The DFT calculation results showed that the 2-EAQ product formed by intramolecular dehydration is free to diffuse out through both straight and sinusoidal channels, however the diffusion of the dimer by-products formed by intermolecular dehydration is restricted. Herein, we firstly propose that the shape-selective catalysis of mild HNO_3_ dealuminated H-Beta zeolite determines its superior performance in E-BBA dehydration. Therefore, through the dual adjustment of acidity and porosity, dealuminated H-Beta has a promising future in the green synthesis of 2-EAQ.

## Conflicts of interest

There are no conflicts to declare.

## Supplementary Material

RA-008-C7RA13576A-s001
