# Supplementary material for: Engineering the porosity and acidity of H-Beta zeolite by dealumination for the production of 2-ethylanthraquinone via 2-(4′-ethylbenzoyl)benzoic acid dehydration
Source: RSC Adv. 2018 Mar 8;8(18):9731–40. doi: 10.1039/c7ra13576a (PMC9078711; doi:10.1039/c7ra13576a)
Supplement: RA-008-C7RA13576A-s001 [file RA-008-C7RA13576A-s001.pdf]

Electronic Supplementary Material (ESI) for RSC Advances.
This journal is © The Royal Society of Chemistry 2018

Engineering porosity and acidity of H-Beta zeolite by dealumination for the production of 2-ethylanthraquinone *via* 2-(4'-ethylbenzoyl) benzoic acid dehydration

J. X. Liu[a], N. He[a], C. Y. Liu[a], G. R. Wang[a], Q. Xin[b] and H. C. Guo[a*]

[a] State Key Laboratory of Fine Chemicals, School of Chemical Engineering, Dalian University of Technology, Dalian 116024, China

[b] State Key Laboratory for Catalysis, Dalian Institute of Chemical Physics, Chinese Academy of Sciences, Dalian 116023, China

Content

Figure S1 .... **S2**

Figure S2 .... **S3**

Figure S3 .... **S4**

Figure S4 .... **S5**

Scheme S1 .... **S6**

Scheme S2/Table S1. .... **S7**

Table S2 .... **S8**

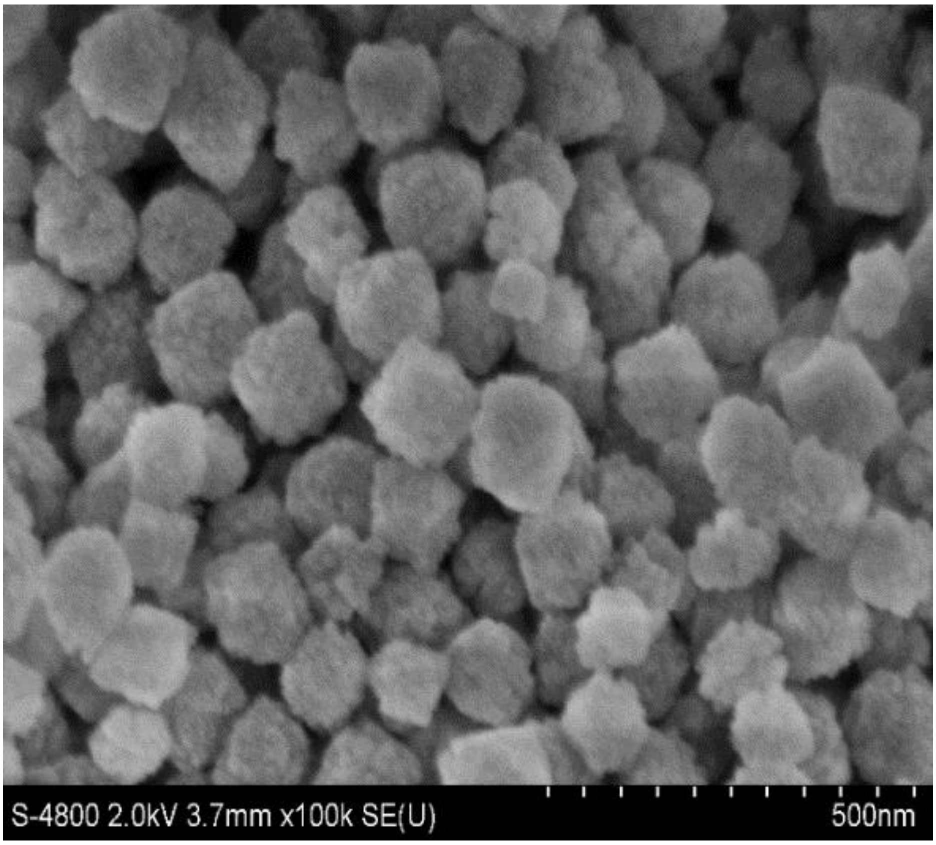

Fig. S1 SEM image of nano-sized H-Beta zeolite.

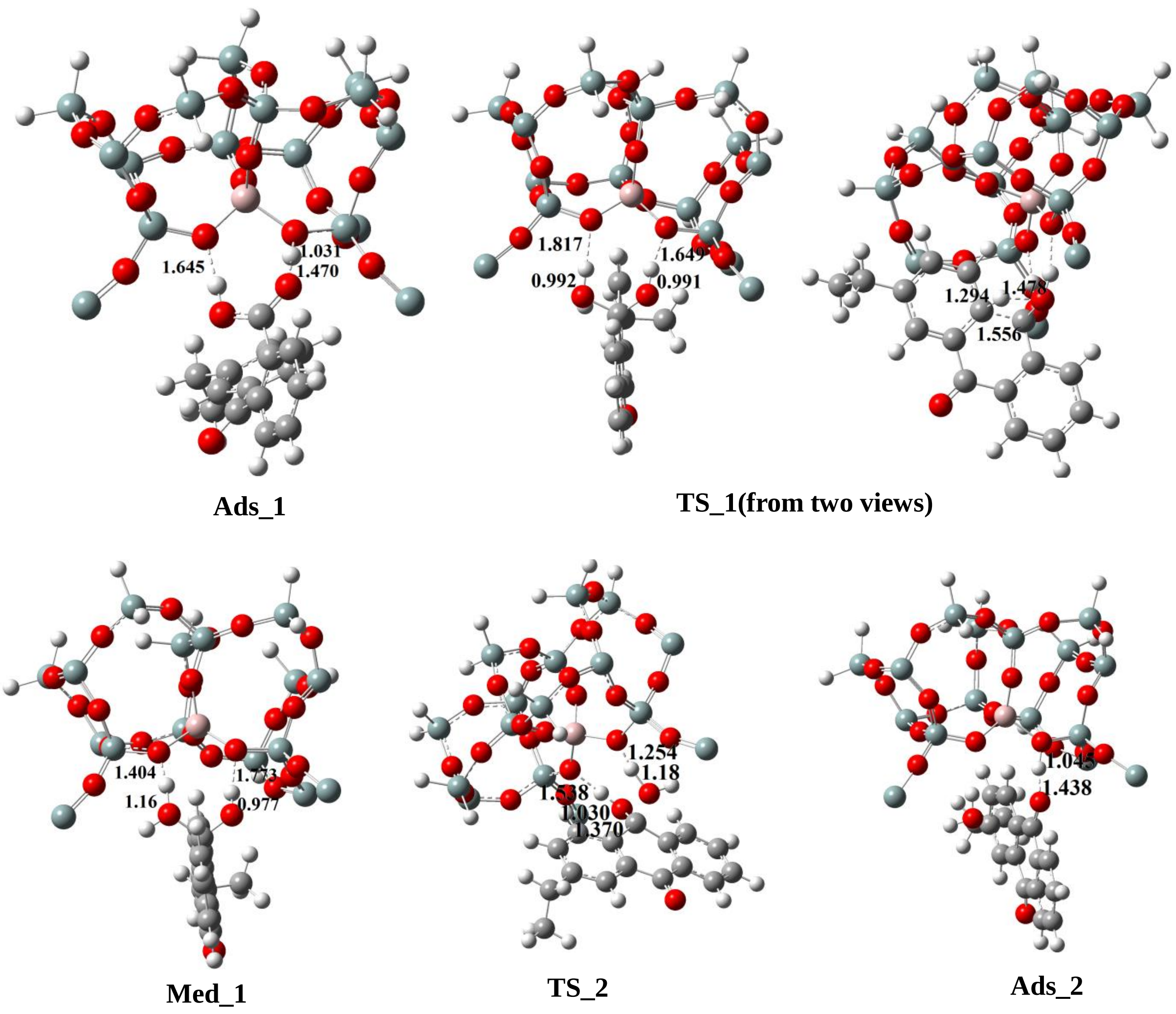

Fig. S2 The structures of E-BBA dehydration over H-Beta zeolites.(Considering the clarity of structures, only the part of structures treated by high-level functional were shown, the others were omitted)

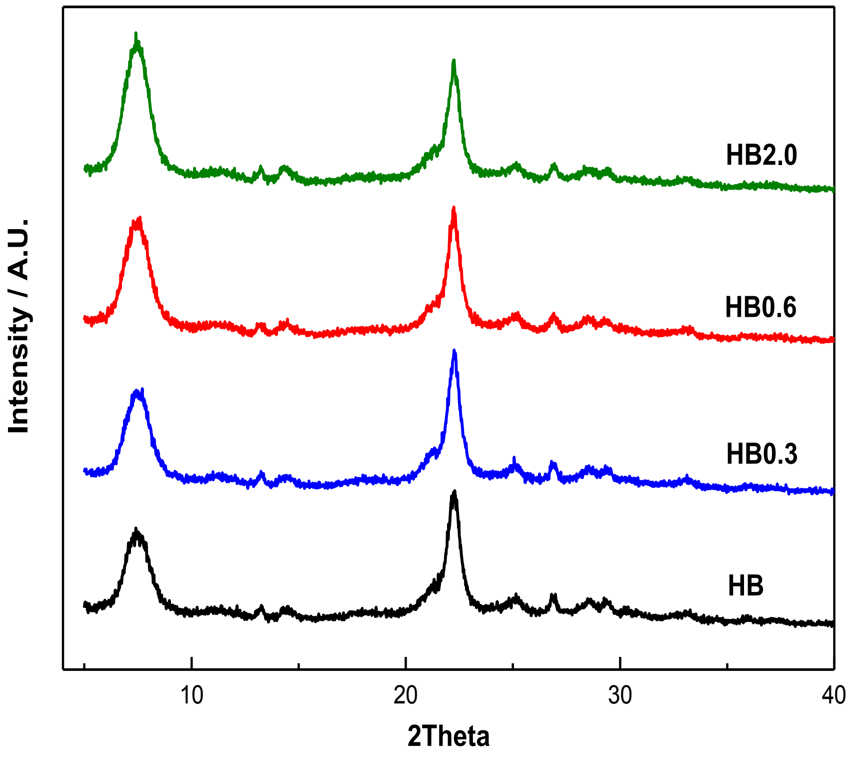

Fig. S3 XRD patterns of H-Beta and dealuminated H-Beta zeolites.

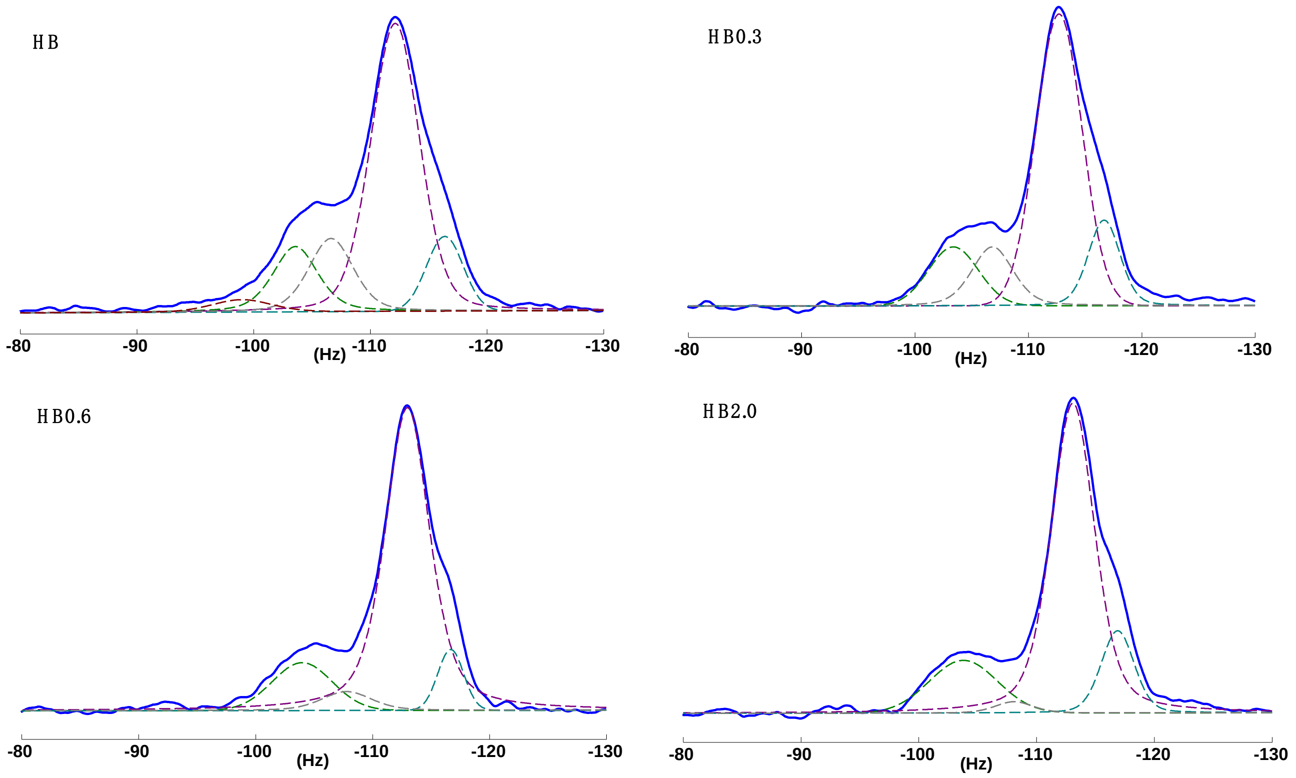

Fig. S4 ^{29}Si MAS NMR spectra of H-Beta and dealuminated H-Beta zeolites.

In the ^{29}Si MAS NMR spectra, five peaks assigned to Q4 (-112 and -116 ppm), Q3 (-107and -103 ppm), and Q2 (-99 ppm) sites were detected[1]. The two peaks for Q4 sites originate from the two different stacking orders polymorph A and polymorph B known for zeolite BEA. The Q3 peaks at -103 and -107 ppm originating from Si(OSi)3(OH)1 and Si(OSi)3(OAl)1 tetrahedrons, respectively. All peaks were fitted with Gaussian functions, and the framework Si/Al ratio is calculated according to literature[2].

References:

[1] Pérez-Pariente J., Sanz J., Fornés V., Corma A., J. Catal., 1990,124, 217.

[2] M.A. Camblor, A. Corma, S.Valencia, Micropor. Mesopor. Mater., 1998, 25, 59.

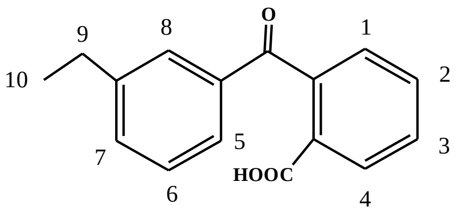

Sites	E (a.u.)	ΔE (kcal/mol)
1	-1610.7933094	0
2	-1610.7917244	0.99
3	-1610.7958588	-1.60
4	-1610.7886260	2.94
5	-1610.7831944	6.35
6	-1610.7910402	1.42
7	-1610.8010084	-4.83
8	-1610.7956514	-1.47
9	-1610.7885487	2.99
10	-1610.7944923	-0.74

Scheme S1 Assumed intermolecular dehydration of the carboxyl of one 2-(4´-ethylbenzoyl)-benzoic acid (E-BBA) molecule with the hydrogen atoms of another E-BBA molecule in positions 1-10, and the energies for all possible products of intermolecular dehydration

In order to make the calculated results comparable, the same method as the high layer of ONIOM was used (ωB97XD/6-31+G(d,p)) to get the energies and structures of two BEA molecules intermolecular dehydration. The energies of all possible species involved in the inter-molecular dehydration have been listed in Table. And only one product, which dehydrated at No.2 position, may fit in the channel of β zeolite. It was shown in following Figure (named as Dimer-1).

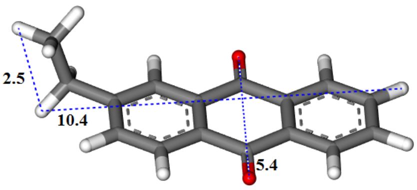

2-EAQ

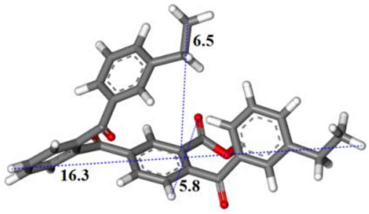

Dimer-1

Scheme S2 Theoretical calculations of the configurations of representative products generated from intra- and inter-molecular dehydrations of E-BBA.

Table S1 Micropores trafficability of H-Beta zeolite for 2-EAQ and dimer-1 of inter-molecular dehydrations*

Micropore sizes in H-Beta zeolite	Straight channels in tetragonal structure, 0.68 × 0.73 nm	Straight channels in monoclinic structure, 0.60 × 0.73 nm	Sinusoidal channels in both structures, 0.55 × 055 nm
2-EAQ size, nm 0.25×0.54×1.04	Yes	Yes	Yes
Dimer-1 size, nm 058×0.65×1.63	Yes	Yes	No

* The micropore sizes of BEA zeolite are crystallographic free diameters; the molecular sizes of 2-EAQ product from intra-molecular dehydration and dimeric by-product from inter-molecular dehydration are calculated by using ONIOM (ωB97XD/6-31+G(d,p)) method.

The theoretically calculated molecular sizes of both intra- and inter-molecular dehydration products of E-BBA are compared with the crystallographic micropore diameters of H-Beta zeolite, it is easy to see that the shape-selective function of the H-Beta zeolite micropores should be a help hand to cripple the inter-molecular dehydration of E-BBA. The direct comparison of these sizes indicates that the micropores of H-Beta zeolite can exclude the formation of most inter-molecular dehydration products. Only dimer-1 is possible to diffuse out through both straight and sinusoidal channels. There is no doubt that, in order to take the advantage of the shape-selectivity of H-Beta zeolite micropores, the external surface of the zeolite should be passivated in advance.

Table S2 Characterization data of H-Beta and dealuminated H-Beta zeolites

Sample	S_{BET}[a] (m^2g^{-1})	V_{pore}[b] (cm^3g^{-1})	V_{micro}[c] (cm^3g^{-1})	V_{meso}[d] (cm^3g^{-1})	c_B[e] ($\mu mol_{Py}\ g^{-1}$)	c_L[e] ($\mu mol_{Py}\ g^{-1}$)
HB	522	0.59	0.08	0.51	111	124
HB0.3	607	0.63	0.06	0.57	107	99
HB0.6	556	0.58	0.08	0.50	60	27
HB2.0	538	0.61	0.05	0.56	4	12

[a] BET method applied to the Ar isotherm.

[b] Volume of Ar adsorbed at p/p_0=0.99.

[c] *t*-plot method applied to the Ar isotherm.

[d] $V_{meso}= V_{pore}- V_{micro}$.

[e] Concentration of Brønsted (c_B) and Lewis (c_L) acid sites derived from the IR study of adsorbed pyridine (Py).

Compared with parent sample HB, the BET surface area increased for all treated samples, but the degree of increased BET surface area was opposite with the concentration of acid, viz., HB0.3>HB0.6>HB2.0. Associated with the NMR results, most of the extra-framework aluminium species at low concentration of acid was eliminated; while further increasing the concentration of acid will dissolve the framework aluminium species which would block the channel again. Therefore, it exhibited in the opposition of degree of increased BET surface area with the concentration of acid.
